# Solvent Separating Secondary Metabolites Directly from Biosynthetic Tissue for Surface-Assisted Laser Desorption Ionisation Mass Spectrometry

**DOI:** 10.3390/md13031410

**Published:** 2015-03-16

**Authors:** David Rudd, Kirsten Benkendorff, Nicolas H. Voelcker

**Affiliations:** 1 Biological Sciences, Faculty of Science and Engineering, Flinders University of South Australia, PO Box 2100, Adelaide, SA 5001, Australia; E-Mail: david.rudd@flinders.edu.au; 2 Marine Ecology Research Centre, School of Environment, Science and Engineering, Southern Cross University, PO Box 157, Lismore, NSW 2480, Australia; 3 Australian Research Council Centre of Excellence in Convergent Bio-Nano Science and Technology, Mawson Institute, University of South Australia, GPO Box 2471, Adelaide, South Australia 5001, Australia; E-Mail: Nico.Voelcker@unisa.edu.au

**Keywords:** secondary metabolites, solvent separation, surface-assisted mass spectrometry, brominated indoles, choline esters, *Dicathaisorbita*, hypobranchial gland

## Abstract

Marine bioactive metabolites are often heterogeneously expressed in tissues both spatially and over time. Therefore, traditional solvent extraction methods benefit from an understanding of the *in situ* sites of biosynthesis and storage to deal with heterogeneity and maximize yield. Recently, surface-assisted mass spectrometry (MS) methods namely nanostructure-assisted laser desorption ionisation (NALDI) and desorption ionisation on porous silicon (DIOS) surfaces have been developed to enable the direct detection of low molecular weight metabolites. Since direct tissue NALDI-MS or DIOS-MS produce complex spectra due to the wide variety of other metabolites and fragments present in the low mass range, we report here the use of “on surface” solvent separation directly from mollusc tissue onto nanostructured surfaces for MS analysis, as a mechanism for simplifying data annotation and detecting possible artefacts from compound delocalization during the preparative steps. Water, ethanol, chloroform and hexane selectively extracted a range of choline esters, brominated indoles and lipids from *Dicathais orbita* hypobranchial tissue imprints. These compounds could be quantified on the nanostructured surfaces by comparison to standard curves generated from the pure compounds. Surface-assisted MS could have broad utility for detecting a broad range of secondary metabolites in complex marine tissue samples.

## 1. Introduction

Secondary metabolites play an important role in the ecological interactions of marine organisms [1], and are fast become a promising source of bioactive compounds with therapeutic potential [2]. Secondary metabolites are produced by species to serve specific functions [1], for example reproduction, predation or defence, and can therefore have heterogeneous expression in tissues both spatially and over time [3,4,5,6]. Traditional solvent separation methodologies are not well adapted for capturing heterogeneously expressed secondary metabolites, requiring either more biological material for recovery [7] or optimized targeted extraction [2], repeated across an ecologically relevant temporal framework.

Yield is a major consideration for both the structural and biological characterisation of secondary metabolites. As metabolites are heterogeneously expressed, determining the optimal tissues and time for extraction can be difficult without some prior knowledge of the biological processes that initiate biosynthesis and storage. Therefore, there is a major benefit in direct analysis of biosynthetic tissue responsible for secondary metabolite production, which can help overcome some of the problems associated with obtaining sufficient bioactive material for isolation and characterisation [7].

Although not involved in primary metabolism, secondary metabolites lie within a complex and abundant mixture of primary metabolites, and their precursors are often synthesised from dietary origins [3]. Therefore, discerning secondary from primary metabolites in tissue can be challenging. Recent bioactive discoveries are showing a trend towards low molecular weight compounds [8,9], placing many secondary metabolites in the same molecular weight range as primary metabolites including adenylates, nucleotides, fatty acids and monosaccharides [10]. Secondary metabolites can also be in very low abundance compared to primary metabolites [2], thus requiring extraction enrichment for detection. Structural features can also help discern secondary from primary metabolites and there are many examples of unique structures in recent discoveries that demarcate them from primary metabolites [2]. Marine secondary metabolites in part have a higher probability of halogenation than terrestrial natural products [11]. Methods that can accurately map molecular species in the low molecular mass range, and are tolerant of heterogeneous marine tissue, will enhance spatial analysis and contribute to our understanding of biosynthesis and function. Spatial analysis and biosynthesis can act as a guide for extraction and subsequent studies on bioactivity. Although there are many strategies for the qualitative, quantitative and spatial analysis of secondary metabolites, the chosen methods must be amenable to the particular tissue being analyzed and be suitable for the accurate detection of the metabolite size range and structural features of interest.

Mass spectrometry imaging (MSI) has emerged as a sophisticated platform for the spatial analysis of proteins, peptides, lipids and small mass metabolites *in situ*. Depending on sample preparation, MSI has the ability to detect thousands of molecular signals simultaneously, reflecting the complexity of biological samples. Across all the mass imaging techniques, matrix-assisted laser desorption ionisation (MALDI)-MSI is the most frequently utilised [12] of the laser desorption ionisation (LDI) methods. Unfortunately, MALDI-MSI has limitations in the low mass range, due to interference from spectra generated by the applied matrix [13]. The use of matrix in the analysis of low molecular weight metabolites complicates data and further challenges the identification of secondary metabolites. MSI also generates considerable spectral data requiring molecular identification, which is a major hurdle in MSI workflows [12], often complicated by matrix ion signals.

New technologies using surface-assisted LDI methodologies [14] have been developed as an alternative to the use of matrices. Nanostructured surfaces have emerged that enhance the detection of small molecules, with an emphasis on increased sensitivity, minimal background signal and simplified sample preparation [15]. Ionization techniques from nanostructured surfaces, such as, nanostructure-assisted laser desorption ionization (NALDI) [14] and desorption ionization on silicon (DIOS) [16,17,18] offer improved mass analysis in the low molecular weight range. Nanostructured surfaces eliminate the need for a matrix as the physical properties of the surface allow effective absorption of the UV laser light, thereby transferring energy to the analyte of interest [16,17]. The properties of the nanostructured surfaces also has a considerable advantage for some tissue types, specifically mucus rich biosynthetic tissues [19]. Functionalization of the DIOS surface attract small molecule metabolites through hydrophobic effects or specific molecular recognition [20,21], which allows other contaminants, like salts and mucus aggregate clumps, to wash away from the area of LDI-MS analysis [19]. NALDI bears some of the same attractive properties as DIOS surfaces, but requires less laser energy for ionization, greatly limiting fragmentation [22]. Nanostructured surfaces provide a matrix-free strategy within the LDI suite of methods when generating spectra, as an alternative to using other direct methods for non-matrix analysis, e.g., ambient methods like direct electrospray ionization mass spectrometry (ESI-MS) in the form of liquid surface extraction analysis (LESA^®^), desorption electrospray ionization (DESI) and nano-desorption electrospray ionization (nanoDESI) [23].

**Figure 1:**
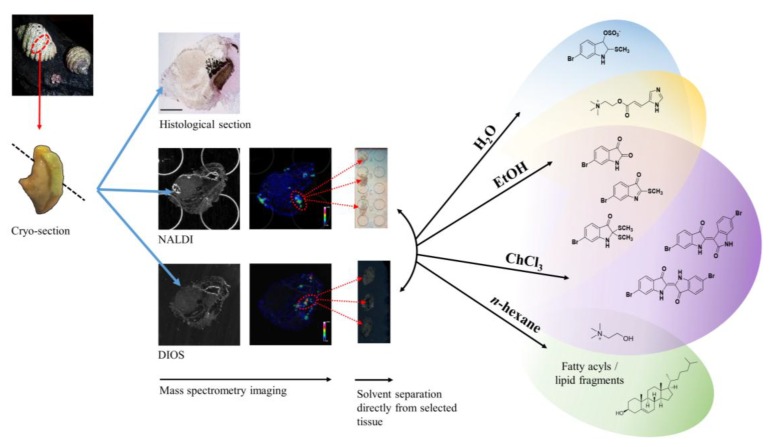
Concept of “on-surface” solvent separation to concentrate secondary metabolites of various polarity from tissue imprints of the biosynthetic regions of *D. orbita*, onto nanostructure-assisted laser desorption ionisation (NALDI) and desorption ionisation on porous silicon (DIOS) surfaces for Mass spectrometry imaging (MSI).

**Figure 2:**
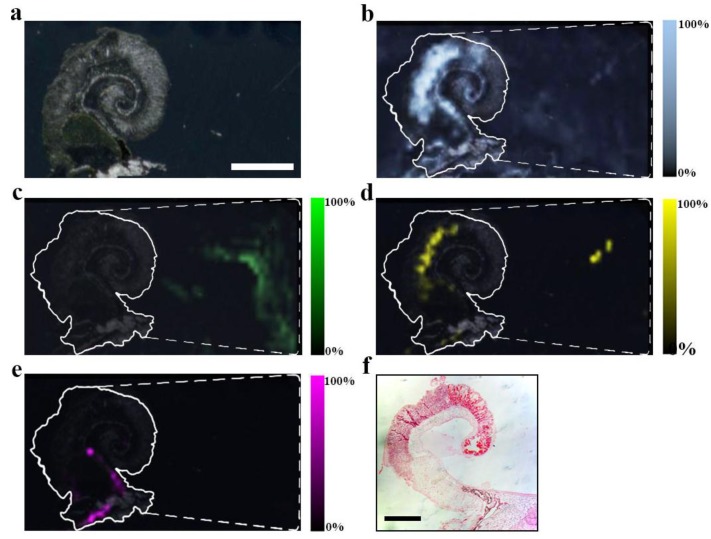
MSI detection region after EtOH separation of secondary metabolites on a DIOS surface. 15 μm thick tissue sections were imprinted onto the nanostructured surface then washed by gently pipetting solvent over the imprint area, onto a clean section of the surface (-----), where the solvent was then allowed to evaporate before LDI-MS; (**a**) scan of tissue on DIOS; (**b**) MSI of murexine 224 *m*/*z*; (**c**) MSI of tyrindoxyl sulfate 340 m/z; (**d**) MSI of tyrindoleninone 256 *m*/*z*; (**e**) MSI of Tyrian purple 421 *m*/*z* and (**f**) example an histological tissue section of the hypobranchial region stained with haematoxylin and eosin.

Recently, we applied NALDI-MSI and DIOS-MSI to detect the spatial distribution of mollusc secondary metabolites *via* a tissue imprinting approach [19]. This approach allowed us to map the distribution of Tyrian purple and its precursors, but the elucidation of some spatially interesting spectra became challenging due to the complexity of spectral signals from the imprinted heterogeneous tissue samples. Spectral data from fragments of larger more labile molecules can be found within the low mass region, making identification of known secondary metabolites difficult and the interpretation of unknown or unexpected secondary metabolites complex. In order to improve secondary metabolite detection and elucidation, a simple chromatographic separation scheme was devised to selectively extract metabolites directly from the tissue on to the nanostructured surface for analysis. Direct separation onto the nanostructured surfaces can be done from frozen sections and under nitrogen gas (Figure 1), reducing enzymatic changes and atmospheric oxidative degradation, while maintaining the benefits of the nanostructured surfaces. Since LDI-MS analysis is also conducted in a high vacuum environment without light, oxidative and photo catalytic degradation affecting less stable secondary metabolites is reduced. The solvent wash area adjacent to the tissue (Figure 2) can subsequently be used to concentrate particular subsets of compounds according to their solubility, thus facilitating identification based on simpler spectra and structural features, e.g. polarity, halogenation. Furthermore, this technique can enable quantification of natural products from tissue imprints by generating standard concentration curves from the purified compounds on the adjacent nanostructured surfaces for comparison of the LDI-MS signal intensity.

Tyrian purple, brominated indole precursors and choline esters have previously been detected in the hypobranchial gland, reproductive structures and egg capsules [5,19,24] of muricid molluscs. The choline esters have potent neuromuscular blocking activity [25,26] and have been implied to aid predation, whilst the brominated indoles are suggested to provide antimicrobial defence in the egg capsules [26,27]. In additional to their ecological value, the choline esters have undergone human clinical trials for pain management [25], whereas the brominated indoles, tyrindoleninone and 6-bromoisatin, have shown promise as anti-cancer compounds, inducing an apoptotic response in a range of cell lines and *in vivo* models [28,29,30,31]. Understanding the distribution of these two classes of secondary metabolites, and the optimization of rapid detection methods, will not only contribute to the ongoing ecological research into their functional ecology, but could also facilitate biodistributional studies in future *in vivo* animal models for development of muricid hypobranchial gland extracts as natural medicines [32].

Here we describe the use of “on-surface” solvent separation of secondary metabolites from tissue imprints of the biosynthetic regions of a marine mollusc (Figure 1). This process allowed solvent extraction of subsets of secondary metabolites, according to polarity, directly from hypobranchial tissue onto nanostructured surfaces for immediate LDI-MS to gain qualitative and relative quantitative data that is compatible with LDI-imaging data [19]. Water (H_2_O), ethanol (EtOH), chloroform (CHCl_3_) and *n*-hexane were independently applied to tissue, to cover a typical solvent profile able to capture hydrophilic to lipophilic low molecular weight secondary metabolites (Figure 1). The identification of particular secondary metabolites that were either retained in the tissue imprints, or washed onto the nanostructured surfaces after solvent separation (Figure 2), was confirmed by standard extraction methods and solvation characteristics, e.g., LogP. Relative quantification of secondary metabolites on the nanostructured surfaces was determined against available standards for 6-bromoisatin, tyrindoleninone and Tyrian purple. Nanostructured surfaces provide an excellent platform for spatial analysis of secondary metabolites [33] using LDI and simple chromatographic manipulations can greatly simplify annotation and de-replication efforts [34]. Direct solvent extraction onto nanostructured surfaces from biosynthetic hypobranchial tissue regions allows rapid and simplified quantitative and qualitative analysis of both brominated indoles and choline esters in the absence of spectra that would otherwise suppress detection.

## 2. Results and Discussion

**Figure 3:**
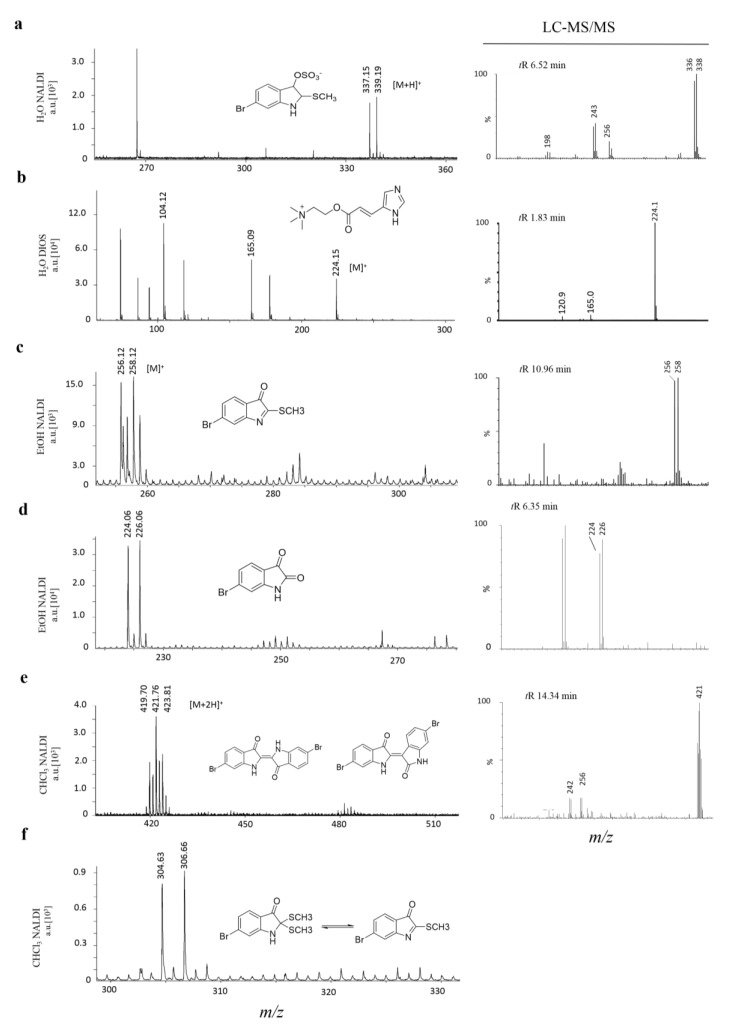
Mass spectra from solvent wash area directly adjacent to hypobranchial tissue. Secondary metabolites on either NALDI or DIOS substrates with adjacent LC-MS/MS of medial hypobranchial extract. Metabolites include (**a**) tyrindoxyl sulfate; (**b**) murexine; (**c**) tyrindoleninone; (**d**) 6-bromoisatin; (**e**) Tyrian purple 6,6′-dibromoindigo (may include isomer 6,6′-dibromoindirubin) and (**f**) tyrindolinone.

On surface solvent extraction from biosynthetic regions of marine mollusc tissue (Figure 1) was effective in detecting spectral signals associated with secondary metabolites on both NALDI and DIOS substrates (Table 1). This approach not only simplified identification (Figure 3) and but also allowed specific metabolite fragments to be co-detected in the absence of complicated spectral patterns associated with primary metabolites and lipid fragments (e.g., murexine, Figure 3). Known metabolites had comparable masses for both NALDI-MS and DIOS-MS, but varied in +H additions compared to LC-MS (Figure 3). Using matrix free LDI-MS, the metabolites were all detected using positive ionization in reflectron mode. High resolution for detected compounds could effectively discern isotopic patterns for identification of bromine. Only two known precursor brominated indoles were not detected using nanostructured surfaces; tyrindoxyl and tyriverdin remained elusive, most likely due to rapid oxidation [32,35].

**Table 1:** NALDI-MS and DIOS-MS detected secondary metabolites from the nanostructured surfaces adjacent to the hypobranchial gland after solvent separation. + Indicates detection within combined spectra.

Compound Class/Compound	Formula	MW	Detected in Solvent Wash				LDI-MS	Major Ions *m*/*z*		Major Ions *m*/*z*	
			Water	Ethanol	Chloroform	Hexane	[M]	NALDI	DIOS	RT (min)	LC-MS
Brominated indoles											
Tyrindoxyl sulfate	C_9_H_7_BrNO_4_S_2_^−^	337.196	+	+			[M + H]^+^	337, 339	337, 339	6.52	336, 338
							[M + 4H]^+^	341, 343	341, 343		
Tyrindoxyl	C_9_H_8_BrNOS	258.140									
Tyrindoleninone	C_9_H_6_BrNOS	256.124		+	+		[M]^+^	256, 258	256, 258	10.96	256, 258
Tyrindolinone	C_10_H_10_BrNOS_2_	304.234		+	+		[M]^+^	304, 306	-		
6-Bromoisatin	C_8_H_4_BrNO_2_	226.029		+	+		[M]^+^	224, 226	224, 226	6.35	224, 226
Tyriverdin	C_18_H_14_Br_2_N_2_O_2_S_2_	514.264								11.67	511, 513, 515
Tyrian purple (6,6′-Dibromoindigo)	C_16_H_8_Br_2_N_2_O_2_	420.060			+		[M + 2H]^+^	419, 421, 423	419, 421, 423	14.34	417, 419, 421
6,6′-Dibromoindirubin	C_16_H_8_Br_2_N_2_O_2_	420.060			+		[M + 2H]^+^	419, 421, 423	419, 421, 423		
Choline esters											
Choline	C_5_H_14_NO^+^	104.173	+	+		+	[M]^+^	104	104		
Murexine	C_11_H_18_N_3_O_2_^+^	224.284	+	+			[M]^+^	224	224	1.83	224

**Figure 4:**
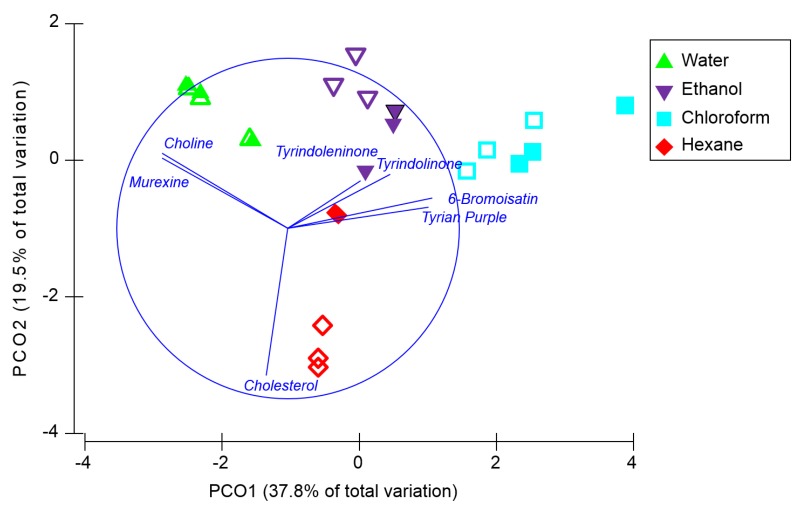
Principal coordinate analysis based on the normalised relative intensities of secondary metabolites detected after solvent separation on NALDI (open symbols) and DIOS (filled symbols) surfaces, grouped according to the type of solvent used (*n* = 3). Trajectory overlay is based on Pearson correlations (*r* > 0.3).

Permutational multivariate analysis (PERMANOVA) revealed that the overall composition of secondary metabolites (Figure 4) varied according to both the type of nanosurface (Pseudo F = 5.5, *p* = 0.004) and the solvent used for on-surface separation (Pseudo-F = 23.5, *p* = 0.001) and there was a significant interaction between these factors (Pseudo F = 4.7, *p* = 0.001). Pair-wise tests revealed that there was no significant difference in the secondary metabolite composition detected by DIOS and NALDI after separation in water (*p* = 0.97) or chloroform (*p* = 0.27), however, the type of nanosurface did influence the compound composition after separation in ethanol (*p* = 0.006) and hexane (*p* = 0.0008) (Figure 4). Irrespective of whether DIOS or NADLI was used, the composition of compounds detected was significantly different between every pair of solvents (*p* < 0.05). Principal coordinate ordination with trajectory overlay based on Pearson correlation (Figure 4) confirms that differences between solvents are driven by polarity, with a strong correspondence between the polarity of the solvent and the hydrophobicity of the compounds (Log *P*, Table 2). These results imply that the type of nanosurface used for MSI and any solvent used for washing the surface after tissue imprinting should be optimised according to the polarity of the secondary metabolites of interest.

**Table 2:** Mean ion intensity (*n* = 3) for secondary metabolites in the solvent wash area after separation from the hypobranchial gland tissue imprints on NALDI and DIOS nanostructured surfaces with corresponding LogP values (calculated using chemo-informatics software ChemBioDraw Ultra 13.0). Significant differences in the ion intensity for each compound were tested using univariate two factor PERMANOVA, followed by pairwise-tests.

Compound	Log *P*	NALDI				DIOS			
		Water	Ethanol	Chloroform	Hexane	Water	Ethanol	Chloroform	Hexane
Choline	−4.236	78,500 (±49,374) ^a^	25,067 (±16,050) ^b^	N.D.	19,500 (±7378)	79,000 (±46,776) ^a^	19,500 (±7378) ^b^	N.D.	6264 (±2139) ^c^
Murexine	−3.373	33,000 (±3812) ^a^	5320 (±1959) ^b^	N.D.	N.D.	33,067 (±3782) ^a^	1450 (±704) ^b^	N.D.	N.D.
Tyrindoxyl sulfate **	−0.346	2625 (±711) ^a^	22,133 (±2108) ^b^	N.D.	N.D.	1809 (±135) ^a,c^	5651 (±3922) ^c^	N.D.	N.D.
6-Bromoisatin *	1.615	N.D.	360 (±213) ^a^	1775 (±725) ^b^	N.D.	N.D.	656 (±646) ^a^	3678 (±485) ^c^	N.D.
Tyrindoleninone *	2.889	N.D.	2506 (±1736) ^a^	2945 (±1277) ^a^	N.D.	N.D.	7957 (±2704) ^b^	2581 (±1311) ^a,b^	N.D.
Tyrindolinone	2.999	N.D.	424 (±107)	958 (±233)	N.D.	N.D.	N.D.	373 (±647)	N.D.
Tyrian purple (6,6′-Dibromoindigo	4.47	N.D.	N.D.	6447 (±2093)	N.D.	N.D.	N.D.	10848 (±4118)	N.D.
6,6′-Dibromoindirubin)									
Cholesterol	7.11	4722 (±755)	N.D.	N.D.	N.D.	N.D.	N.D.	N.D.	N.D.

Different letters ^a, b & c^ indicate significant differences between each nanosurface and solvent combination (*p* < 0.05); Overall differences between the ion intensity on NALDI and DIOS are also indicated for each compound as *p* ≤ 0.05 (*) and *p* ≤ 0.01 (**); N.D. = not detected.

### 2.1. Hydrophilic Compounds

H_2_O extraction from the medial hypobranchial gland was effective in concentrating the hydrophilic compounds tyrindoxyl sulfate, choline and choline ester murexine, directly onto the mass spectrometry surfaces (Figure 3, Table 1). Both NALDI-MS and DIOS-MS detected ions at 337, 339 *m/z*, corresponding to the [M + H]^+^ ion for tyrindoxyl sulfate (Figure 3). Interestingly, tyrindoxyl hydrogen sulfate, [M + 4H]^+^, was detected in the tissue and immediately adjacent to the tissue for DIOS-MS only. The [M + H]^+^ for tyrindoxyl hydrogen sulfate was detected on both substrates, but further out from the tissue margin, 10 to 20 mm away (e.g., Figure 2c).

NALDI-MS and DIOS-MS consistently detected peaks at 224.1 *m/z*, the [M]^+^ ion for murexine, up to 20 mm away from the tissue margin. The assignment for murexine is further supported by the fragment ion at 165 *m/z*, seen co-located with the parent ion (Figure 3). Cleaner spectra afforded by solvent separation for murexine makes the assignment of ions and fragment ions easier due to the consistency in the co-localized peaks, which is comparable to collision induced dissociation (CID) fragment ions from LC-MS/MS (Figure 3). Choline was also detected co-localized with murexine using both surfaces.

Choline was the most dominant ion detected from H_2_O separation (Table 2), followed by murexine (mean intensity ~33,000 a.u. for both surfaces; Table 2). Tyrindoxyl sulfate was also detected by H_2_O separation, but at less than one tenth of the intensity of murexine (Table 2). Tyrindoxyl sulfate forms a salt complex with both choline ester murexine and choline in the hypobranchial gland [36]. This is considered to be a mechanism by which these secondary metabolites are stored prior to liberation from the medial region into the external environment [32]. The detection of tyrindoxyl hydrogen sulfate [M + 4H]^+^ within and immediately adjacent to the tissue may be an indication of the salt of choline ester complex, whereas the [M + H]^+^ of tyrindoxyl sulfate could be the liberated compound, which undergoes enzymatic cleavage by an arylsulfatase enzyme [37]. Murexine is a natural tranquiliser secreted by predatory species of the Muricidae, and has been suggested to be a predatory specialization due to its absence in herbivorous or scavenging molluscs [26].

Washing is often a part of MSI workflows as a means of removing residual salt during sample preparation. As demonstrated with the H_2_O solvent separation here, excessive washing could lead to delocalisation of hydrophilic secondary metabolites and a complete loss of detection (Figure 2). Previous MSI of the hypobranchial region show tyrindoxyl sulfate is still detected and largely remains localised to the medial hypobranchial gland [19], an expected location, but can be completely washed out of tissue if using enough solvent (Figure 2).

### 2.2. EtOH Solvent Separation

Signals for tyrindoxyl sulfate [M + H]^+^, choline and murexine were detected on both substrates after EtOH separation from the tissue (Table 1). EtOH also removed tyrindoleninone 256, 258 *m/z* [M]^+^; tyrindolinone 304, 306 *m/z* [M]^+^; and 6-bromoisatin 224, 226 *m/z* [M]^+^, detected across the entire solvent area 20 mm from the tissue margin (Figure 3, Table 1). Tyrindoxyl sulfate, was detected only as the [M + H]^+^ ion and showed a significantly higher mean intensity on NALDI than DIOS surfaces (*p* = 0.0026). In comparison to H_2_0 separation, the mean intensity of tyrindoxyl sulfate had an almost tenfold increase in intensity in the ETOH residue on NALDI-MS (*p* = 0.001, Table 2). Conversely, on-surface detection of murexine after EtOH separation resulted in a significant six times reduction in mean intensity, compared to H_2_O separation from both NALDI and DIOS surfaces (*p* = 0.007, Table 2). Choline intensity was also significantly reduced after EtOH separation (*p* = 0.04, Table 2). Of the solvents tested, H_2_O is ideal for the extraction of the cationic murexine and choline, whilst EtOH extraction maximizes recovery of the counter ion tyrindoxyl sulfate.

Tyrindoleninone was detected at a significantly higher intensity on DIOS-MS compared to NALDI-MS and DIOS after EtOH separation (*p* = 0.045, Table 2), whereas tyrindolinone was only detected on NALDI-MS at a low intensity (Table 2). Tyrindoleninone, tyrindolinone and 6-bromoisatin are intermediate indoles which form after the enzymatic cleavage of tyrindoxyl sulfate [35] by an arylsulfatase enzyme [37]. Intermediate brominated indoles are formed by oxidative and photolytic degradation, which leads to variability in their relative quantities in tissue samples. This variability can be minimized across samples by careful handling, particularly by minimizing exposure to light and oxygen. The major point at which samples are most exposed to light and oxygen during on-surface solvent separation is during the placement of sections onto the nanostructured surface. Degradation was further reduced using a gentle stream of nitrogen gas when handling. Outside of sample placement, degradation can be minimized as tissue can be sectioned frozen (held at −80 °C and brought down to −20 °C in the cryostat during cutting), and LDI-MS is done in a high vacuum with no light. Therefore, LDI-MS detection should be done immediately after solvent separation to prevent on-surface degradation of precursors that have separated from the tissue.

The differences in measured intensity of some secondary metabolites between the two surfaces could be attributed to the differences in the surface chemistry. NALDI plates are surface coated with a layer of inorganic nanostructures to absorb laser energy (Bruker Daltronics), whilst the pSi are functionalised with silanes (F5PhPr) and the etched porosity provides laser energy absorption preventing excessive fragmentation from the laser shot [20]. In this case, the use of ethanol solvent separation shows NALDI to desorb and ionise hydrophilic compounds better then DIOS. This indicates that DIOS may be more hydrophobic than NALDI surfaces. The advantage of DIOS, over NALDI, is that DIOS can be treated with different silanes [20], which can change the hydrophobicity of the surface to target specific classes of secondary metabolites.

### 2.3. CHCl_3_ Solvent Separation

Both NALDI-MS and DIOS-MS failed to detect the more hydrophilic compounds after CHCl_3_ separation, as anticipated (Table 2). Tyrindoleninone, tyrindolinone and 6-bromoisatin were detected on both substrates across the solvent area. CHCl_3_ also effectively extracted Tyrian purple with detected spectra showing a triplet cluster centred at 421 *m/z* [M + 2H]^+^ (Figure 4e) on both NALDI and DIOS substrates. CHCl_3_ has been the solvent of choice when extracting brominated indoles from fresh macerated hypobranchial glands [28,29,30] and NALDI-MS and DIOS-MS detected all expected metabolites except tyrindoxyl and tyriverdin [35]. Tyrindoxyl is an unstable intermediate created by enzymatic cleavage of tyrindoxyl sulfate with an arylsulfatase enzyme [35], which is also expressed in the medial hypobranchial gland [37]. The absence of tyrindoxyl from LDI analysis could be due to the unstable nature of the compound. The absence of tyriverdin from both substrates, indicated by the lack of the parent ion calculated as a triplet ion cluster centred on 514 *m/z*, may be due to the high instability of this compound, as previously reported from mass spectrometry analyses [5]. Triplet ion clusters, characteristic of di-brominated compounds, were detected with centre peaks at 483 and 438 *m/z*, which may be fragment ions of tyriverdin.

6-Bromoisatin was detected at significantly higher intensities on DIOS-MS compared to NALDI-MS (*p* = 0.0157) and showed a significant five-fold increase after CHCl_3_ separation in comparison to EtOH (*p* = 0.0005, Table 2). Tyrian purple was only detected in CHCl_3_ separations and the mean LDI-MS detection was not significantly different on DIOS compared to NALDI surfaces (*p* = 0.17, Table 2). Therefore, despite the fact that a greater diversity of compounds were detected after EtOH separation, CHCl_3_ increases the detection of some brominated indoles. This could be a result of higher LogP and solvation characteristics that are more suited to CHCl_3_ extraction. Alternatively, as 6-bromisatin and 6,6-dibromoindigo are both end-products in a series of oxidative and photolytic reactions, solubility of intermediate precursors in CHCl_3_ may facilitate increase degradation.

### 2.4. Lipophilic Separation Using n-Hexane

After hexane solvent separation LDI-MS detected a consistent signal at 104 *m/z*, which is the [M]^+^ ion for choline (Table 1 and Table 3). Choline has been detected in *D. orbita* from the medial hypobranchial gland [26] co-localized with choline esters, the natural tranquilizers found in many members of the Muricidae [26]. Choline is also known to be associated with tyrindoxyl sulfate, acting as a counter ion prior to secretion from the tissue [26,36]. The mean intensity of choline was significantly lower after hexane separation in comparison to water (*p* = 0.0172) and ethanol (*p* = 0.0121), as would be expected from its high polarity (Table 2). However, other consistent peaks detected in hexane samples include 184 *m/z*, corresponding to phosphocholine headgroup (Table 3), which is a characteristic fragment ion of phosphatidylcholine lipid groups [38]. The 184 *m/z* ion has been detected during secondary ion mass spectrometry imaging of *Aplysia californica* neurons [38], where it is a fragment from the lipid component of the neural cell membrane.

**Table 3:** Primary metabolites and associated lipophilic ions detected after hexane separation originating from either free fatty acids or phospholipid origin. References indicate previous detection in molluscan tissues.

*m/z*	Formula	[M]	Lipid Match	Reference
86.119				
104.136	C_5_H_14_NO		Choline	[36]
184.101	C_5_H_15_NPO_4_	[M + H]^+^	Phosphocholine headgroup	[38]
198.114				
228.277	C_14_H_36_O_2_	[M]^+^	Myristic acid	[39,40]
256.309	C_16_H_32_O_2_	[M]^+^	Palmitic acid	[39,40]
368.448	C_27_H_44_	[M − OH]^+^	Cholesterol	[39,41]

Consistent ions at 256 *m/z* (Table 3) are most likely generated by the saturated fatty acid, palmitic acid [M]^+^. This is a dominant saturated fatty acid found in the adult tissues of many gastropod species [42] and the benthic encapsulated embryos of gastropods molluscs, including *D. orbita* [39]. Myristic acid, another saturated fatty acid detected in gastropod tissues [42] and gelatinous spawn [39], was assigned based on peaks detected at 228 *m/z*. Fatty acids and choline may also originate from phospholipid fragments, like the 184 *m/z* ion, which can be generated from post source decay [43]. The other remaining consistent peak in the hexane separation on NALDI is likely to be cholesterol, detected as the [M − OH]^+^ at 368.4 *m/z* (Table 2 and Table 3). Cholesterol is known to be a major sterol found in molluscan tissue [40] and the dominant sterol in benthic spawn [39]. Cholesterol can often be found largely intact using mass spectrometry analysis, usually in the [M + H − OH]^+^ form, and has been detected in *Aplysia* neurons [38].

Cholesterol, fatty acids, and lipids more generally, provide a considerable proportion of the fuel reserves within the muscles and organs of adult molluscs [39,41]. Cholesterol is also known to be a major component of the cell membrane, where it interacts with sphingolipids to order the membrane structure, influencing membrane permeability and fluidity [44]. As lipids contribute to fuel reserves in molluscs, lipid-rich tissues or organs adjacent to secretory or storage regions may be an indication of biosynthetic sites where secondary metabolites are constructed prior to storage and deployment. Alternatively, lipid-rich regions in adult females may be sites where fatty acids, cholesterol or specialised lipid structures are incorporated into egg capsules or spawning material. The lipid content of mollusc egg masses have shown antimicrobial activity, which would aid in the viability of benthic egg masses during development [39]. Simplified spatial analysis of maternal derived lipid classes would be an excellent tool for assessing resource partitioning within the field of chemical ecology.

### 2.5. Quantification of Secondary Metabolites Using Nanostructured LDI-MS

**Figure 5:**
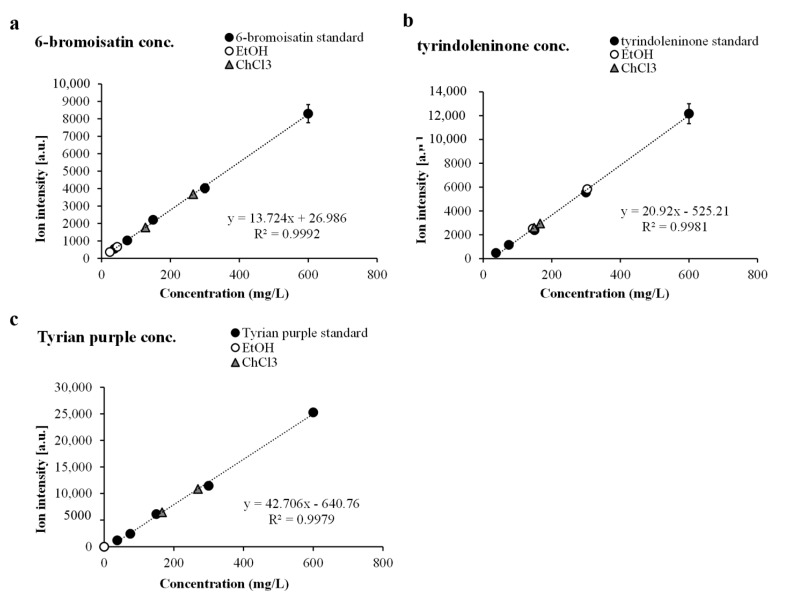
Relative quantity of *ex situ* secondary metabolites from the hypobranchial gland compared to standard curves for (**a**) 6-bromoisatin; (**b**) tyrindoleninone and (**c**) Tyrian purple from triplicate samples at five concentrations.

Quantification using mass spectrometry intensity data requires the adjacent use of known standards or purified compound to establish the level of comparative ion intensity. Available standards applied adjacent to the solvent area allowed the quantification of the highest intensity detected for tyrindoleninone, 6-bromoisatin and Tyrian purple (Table 2) compared to linear concentration curves (Figure 5). Not all metabolites behave in the same fashion during mass spectrometry ionisation/vaporisation or subsequent time-of-flight analysis (TOF) [45]. When using reflectron mode for LDI-MS, which has suitable resolving power in the low molecular mass range, the pulsed laser extraction delay is optimised to allow plume formation and equivalent ion velocities entering into the TOF tube [46]. Lower delayed extraction times create a window for ion selection which produces a bias towards the low molecular mass range [45], reducing resolution and peak intensity for larger compounds. Therefore LDI mass window settings must remain constant during acquisition and intensity values cannot be extrapolated outside of the parameters for each acquisition.

Purified fractions of 6-bromoisatin, tyrindoleninone and Tyrian purple were used effectively to generate standard-curves (Figure 5). This facilitated approximation of metabolite *ex situ* relative quantification, as the increases in intensity follow linear trends (Figure 5). The *ex situ* distribution changes across the solvent wash area (Figure 2), so the highest intensity detected for each compound from the summed spectra was selected for comparison to standard curves. Purified 6-bromoisatin dissolved in DMSO at 600, 300, 150, 75 and 37.5 mg/L applied to both NALDI and DIOS surfaces adjacent to solvent separation wash, produced a linear concentration gradient (*R*^2^ = 0.9941, Figure 5a). Tyrindoleninone also produced a linear concentration gradient (*R*^2^ = 0.959, Figure 5b) when dissolved in DMSO at the same five concentrations. Finally, a purified fraction of Tyrian purple (600, 300, 150, 75 and 37.5 mg/L) gave a linear concentration gradient for LCI-MS signal intensity (*R*^2^ = 0.989, Figure 5c).

Finding the balance between effective ionisation and metabolite fragmentation can be difficult across multiple metabolites, whilst reducing sample-to-sample variability. Compounds like murexine readily fragment at the linear carbon chain, losing the methyl ammonium, in a process called post source decay. Therefore, some compounds are not easily quantifiable. Heterocyclic compounds, like the brominated indoles, experience less post source decay and spectra tend to contain the [M]^+^ or [M + H]^+^ parent ion, with very minor amounts of fragment ions. No sodium or potassium adducts were detected for any of the compounds, which could be considered a major advantage of the nanostructured surfaces compared to other hyphenated MS techniques.

Another interesting quality of the nanostructured surfaces is the way in which samples are deposited in a largely homogeneous concentration. The linear increase in the intensity detected from purified compounds indicates an even concentration across the surface, which is also matched by the solvent separation directly from tissue. This is considered to be one of the attributes of the porous material, in that the surface acts in a “sponge like” manner to trap small metabolites [17]. Traditional MALDI-MS requires careful consideration when applying a matrix for distributional or quantification studies because when matrix solutions dry they form crystal structures which can alter the distribution of the analyte [47]. Poorly applied matrix or the dry drop method for MALDI-MS tends to show laser spot to spot variability, seen as large changes in intensity for the same sample and “sweet spots” when scanning across the sample [47]. The lack of matrix for the nanostructured surfaces reduces this effect.

## 3. Experimental Section

### 3.1. Tissue Preparation for Extraction and Solvent Separation on MSI Substrates

Adult *D. orbita* samples were collected off rocky intertidal coastlines on southern metropolitan rocky reefs in South Australia using an Exemption Permit to the South Australian Fisheries Management Act 2007 section 70 (Permit No. 9902638). The selected adult specimens were prepared by cracking open the shell with a vice at the junction between the primary body whorl and spire and the soft body was removed by cutting the columnar muscle. The soft tissue was then rinsed in MilliQ water to reduce residual salt. Males were identified by the presence of a penis above the tentacles [48] and the absence of egg capsule glands and ovaries. The male hypobranchial glands were removed by incision along the connective mantle tissue between the ctenidium and the branchial hypobranchial and the posterior prostate and digestive gland. The hypobranchial gland and prostate gland were left connected [5] and placed in 5 mL polypropylene sample tubes (Sarstedt, Nümbrecht, Germany) and snap frozen in liquid nitrogen. Frozen tissue samples were protected from light and stored at −80 °C until required.

### 3.2. Extraction of Secondary Metabolites for LC-MS

#### 3.2.1. Extraction and Chemical Analysis of Brominated Indoles

Solvents were purchased from Sigma-Aldrich (CHROMASOLV^®^, HPLC grade, Castle Hill, NSW, Australia). Eight fresh hypobranchial glands (6.58 g) were solvent extracted as previously described [49] to obtain brominated indoles. Briefly, secondary metabolites were extracted from glandular tissue in an equal portion of chloroform and methanol (1:1 v/v, Sigma) and continuously stirred overnight. After vacuum filtering (Whatman filter paper 1), the polar and lipophilic fractions were separated using 20 mL MilliQ water. The chloroform fraction contained the intermediate precursor brominated indoles and Tyrian purple, whilst the methanol fraction contained the hydrophilic, tyrindoxyl sulfate. Each fraction was evaporated to dryness on a Rotavapor^®^ R-114 (BÜCHI Labortechnik AG, Flawil, Switzerland), weighed and re-dissolved in 1 mL of acetonitrile within amber vials for LC-MS analysis.

Brominated indoles in hypobranchial extracts were analyzed by high performance liquid chromatography (HPLC; Waters 2695, Waters Alliance^®^, Rydalmere, NSW, Australia) coupled to a mass spectrometer (MS; Micromass Quatro micro™ tandem quadrupole MS System, Waters, Milford, MA, USA). HPLC separation was performed on a reverse-phase hydrophobic column (Synergi™, Hydro-RP, 4 μm C18 phase, 80 Å, 250 mm × 4.6 mm i.d., Phenomenex, Lane Cove, NSW, Australia) using a gradient of acetonitrile (ACN) in water with 0.1% formic acid detecting extract components with parallel UV/Vis diode-array at 300 and 600 nm [30]. The gradient was applied at a 1 mL/min flow rate starting with 30% ACN for 1 min, 60% for 3 min, and 100% for 15 min before returning to 30% for 15 min. ESI-MS detected brominated indoles at a flow rate of 300 μL/min, in full scan mode, and recorded using the MassLynx 4.1 data system (Waters Alliance). Retention times were standardized using 4 μM synthetic 5-bromoisatin (Sigma-Aldrich, technical grade) in acetonitrile. The identification of brominated indoles was based on peak retention time, expected calculated mass and isotopic clusters for the mono- and di-brominated compounds within mass spectra [30].

#### 3.2.2. Extraction and Chemical Analysis of Choline Ester Murexine

Murexine was extracted from 1.3 g of hypobranchial tissue in three times 30 mL volumes of acetone and pooled. The extract was vacuum filtered through a PTFE membrane filter (pore size 0.2 μm), evaporated to dryness, and washed three times with 10 mL of diethyl ether to remove fats. Total extract was then taken up in 5 mL of ACN for LC-MS.

Murexine was detected by ultra-performance liquid chromatography (UPLC)-MS. Separation was provided by an Acquity UPLC^®^ system (Waters Alliance), using a 10 μL injection volume, on a reverse-phase column (Atlantis T3, 3 μm C18, 3 × 100 mm i.d., Waters Alliance) with a mobile phase of 0.5% formic acid (A) and acetonitrile (B) at a flow rate of 0.5 mL/min (gradient of solvent: 0–10 min, 98% A and 2% B), with parallel UV/Vis PDA detection. ESI-MS detected the murexine structure on the Micromass Quatro micro™ tandem quadrupole mass spectrometer and data was acquired using MassLynx 4.1 data system (Waters Alliance). To see the structural features of murexine in ESI, a scan at 20 V was compared to a collision induced dissociation (CID) scan at 35 V cone voltage (positive ion electrospray, 80 to 500 m/z mass range) for comparison to post source fragmentation that occurs in both NALDI and DIOS-MS.

### 3.3. NALDI and DIOS Surface Fabrication, Oxidation and Functionalization

NALDI substrates were obtained from Bruker Daltronics. DIOS surfaces were fabricated according to [19]. Briefly, monocrystalline (0.008–0.02 Ωcm) antimony doped *n*-type Si (100) wafers (Silicon Quest International, CA, USA) were cut, methanol sonicated for cleaning and dried prior to substrate fabrication by light-assisted anodic etching [19,50]. Silicon substrates were secured using a custom built Teflon cell in contact with a gold foil anode (Space Products International, CA, USA) and a platinum wire cathode (0.5 mm, 99.9%; Aldrich, WA, USA), shaped into a ring. Etching was achieved through the addition of an electrolyte solution 1:1 hydrofluoric acid (HF):EtOH. The submerged pSi surfaces were illuminated using a fiber optic light source passing through a set of two aspheric lenses, *f* = 80 mm (OptoSigma, CA, USA) for collimation. A 20 mA constant current was applied across the Teflon cell for 2 min via a 2425 current source meter (Keithley, Cleveland, Ohio, OH, USA), operated via a meter program constructed in LabView 6.1. Fabricated pSi were washed with methanol prior to being dried under nitrogen gas.

Freshly etched pSi were oxidized using ozone at a flow rate of 3.25 g/h (Ozone-Generator 500, Fischer, Germany). After oxidation, pSi were subjected to a second pore broadening etch with 5% HF/H_2_O for 30 s and re-ozone oxidised. The hydroxyl-terminated pSi surfaces were then silanized using 80 μL of neat silane (F_5_PhPr) for 15 min at 90 °C. Silanized pSi were washed with methanol, dried under nitrogen gas and stored in a desiccator until required.

### 3.4. Tissue Sectioning, Deposition and Solvent Separation on NALDI and DIOS Surfaces

Tissue selection was based on the imaging results generated for NALDI and DIOS-MSI of 15 μm sections of the medial hypobranchial gland from adult male *D. orbita* [19], Figure 1. Fresh frozen glands were mounted and prepared for sectioning according to Ronci *et al.* [19]. Glands were transversely cryo-sectioned until the medial region was exposed. The mounted frozen gland was then rotated to section only the medial tissue region of the hypobranchial gland, allowing only that single region to be collected for depositing onto NALDI and DIOS-MS substrates. Thin 15 μm thick sections were placed on either a NALDI or DIOS surface. Three replicate tissues sections (*n* = 3) were cut for each solvent separation for both NALDI and DIOS surfaces (total *n* = 24). Secondary metabolites were separated out of each tissue section by washing across the tissue onto a clean section of the nanostructured surface adjacent to the tissue, Figure 4, using either 200 μL of H_2_O, EtOH, CHCl_3_ or *n*-hexane for both NALDI and DIOS surfaces. Solvent was applied by gentle pipetting. The solvent was then allowed to evaporate from the surface in a fumehood under a stream of N_2_ gas at room temperature before MS.

### 3.5. NALDI and DIOS-MS Acquisition

NALDI surfaces, post solvent separation, were mounted into a specialized steel adapter target, whilst DIOS surfaces were mounted onto a customized MTP 384 ground steel target plate (Bruker-Daltronics GmbH, Bremen, Germany), secured with conductive carbon tape, and loaded into an Autoflex III TOF/TOF mass spectrometer (Bruker-Daltronics) equipped with a SmartBeam 200 Hz laser. Quadratic external calibration of the TOF tube was performed before each new surface acquisition using α-cyano-4-hydroxycinnamic acid (CHCA) adducts together with bradykinin (1–7) and angiotensin II, spotted on a free area of the surface. Monoisotopic peaks for the calibration range included: K^+^ 38.9637, CHCA [M + H − H_2_O]^+^ 172.0399, CHCA [M + H]^+^ 190.0504, CHCA [M + Na]^+^ 212.0324, CHCA [2M + H − CO2]^+^ 335.1032, CHCA [2M + H]^+^ 379.0930, bradykinin Fragment 1–7 [M + H]^+^ 757.3991 and angiotensin II [M + H]^+^ 1046.5418. Surface calibration was determined based on the brominated indole standards used to generate standard curves.

Samples were run in reflectron positive mode in the 20–1000 Da range, with a medium laser focus, corresponding to a 50 μm diameter. Electronic gain was set to regular. Laser intensity was maintained at 42%, with a 20 ns pulse delay to allow plume formation for low mass molecules. Ion and detector settings were: ion source 1–19.00 kV, ion source 2–16.80 kV, lens–8.25 kV, reflector 1–21.00 kV, reflector 2–9.40 kV. Data sets were further processed by baseline subtraction (1) and normalized noise threshold settings (0.5).

Spectra were acquired up to 20 mm from the margin of the tissue section to evaluate any effect on concentration distribution. At each sampling point a summed spectra was acquired by five 200 pulsed laser shots (1000 shots). Each different surface had a standard reference for purified 6-bromoisatin at 600, 300, 150, 75 and 37.5 mg/L; purified tyrindoleninone at 600, 300, 150, 75 and 37.5 mg/L; and purified Tyrian purple, 6,6′-dibromoindigo at 600, 300, 150, 75 and 37.5 mg/L, all using serial dilution in triplicate.

### 3.6. Mass Spectrometry Imaging

Imprinted pSi chips were mounted onto a customised MTP 384 ground steel target plate (Bruker-Daltronics GmbH, Bremen, Germany), secured with conductive carbon tape, and loaded into an Autoflex III TOF/TOF mass spectrometer (Bruker-Daltronics) equipped with a SmartBeam 200 Hz laser. NALDI plates were mounted onto the NALDI adaptor target and loaded into the Autoflex III. Scanned tissue images, on pSi or NALDI substrates prior to solvent separation, were loaded into FlexImaging 2.1 (build 25) and aligned with the steel target plate containing the pSi or NALDI sample based on three teach points. The imaging area was selected based on the tissue area plus the 20 mm solvent wash area adjacent to the tissue. FlexImaging 2.1 distribution maps were used to control FlexControl 3.3 (build 85) during image acquisition. Samples were run in reflectron positive mode in the 20–1000 Da range, with a spatial resolution of 150 μm and medium laser focus, corresponding to a 50 μm diameter. Colour maps for secondary metabolites were based on the corresponding *m/z* which was evaluated using spot spectra analysed in FlexAnalysis 3.3 (build 65).

### 3.7. Statistical Analyses

Statistical analyses were undertaken using Primer V6 + PERMANOVA add-on. For all analyses, Euclidean distance similarity matrices were generated from the normalised relative intensity values and 9999 permutations were run. A two factor multivariate analysis was used to compare the overall composition of compounds detected on DIOS and NALDI, as well as between the different solvent separations. Two factor univariate PERMANOVs were also used for each individual compound. In cases where a significant interaction was detected between factors, pairwise tests on were run grouped according to the nanostructure, as well as the solvent. Monte Carlo tests were used to establish significance in the cases where ≤10 unique permutations were possible. The multivariate data was also graphically represented using a principle coordinate ordination with vector overlay based on Pearson correlation *r* > 0.3.

## 4. Conclusions

Nanostructured surfaces for use in LDI-MS analysis broaden the qualitative, quantitative and spatial MS analysis of low molecular weight compounds. They are ideal for small molecular weight primary and secondary metabolites because they are not masked or impeded by matrix ions. Nanostructure-assisted LDI-MS provides an effective platform for the spatial analysis of heterogeneous tissue, requiring minimal samples sizes (effective for 4 μm thick samples) and detecting metabolites across multiple structural classes over a wide polarity range. Simple chromatographic separations on NALDI and DIOS surfaces after tissue imprinting can effectively simplify the efforts for annotating LDI spectra. The NALDI and DIOS surfaces also show promise for quantifying known metabolites in tissue samples. The simplified analytical technique combined with the broad utility of the surfaces are a good reason for their adoption in chemical ecology, marine natural products, metabolomics and any field concerned with characterizing metabolites.
